# Supplementary material for: Growth‐ and stress‐related defects associated with wall hypoacetylation are strigolactone‐dependent
Source: Plant Direct. 2018 Jun 13;2(6):e00062. doi: 10.1002/pld3.62 (PMC6508513; doi:10.1002/pld3.62)

A

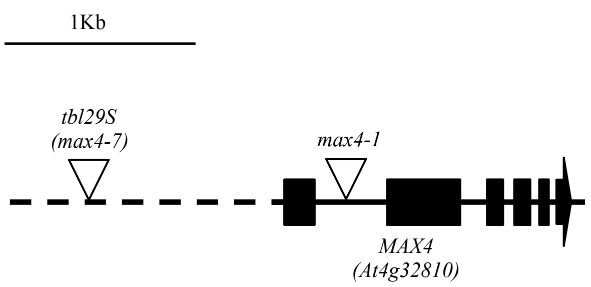

Supp. Figure 1. *tbl29S* mutation represents an insertion into the At4g32810 promoter region.

(**A**) Genomic model of *MAX4* (At4g32810) . Black boxes indicate coding exons. Open triangles mark the positions of the *max4-1*(CS9568 stock number) and *max4-7* (*tbl29S*) insertions.

(**B**) *MAX4* gene expression in Col-0, *tbl29* and *tbl29*-suppressed plants. Data are represented as mean (AVG) ± the standard deviation (SD) of 3 biological replicates. Means with different letters are significantly different (Tukey's HSD, $p<0.05$)

B

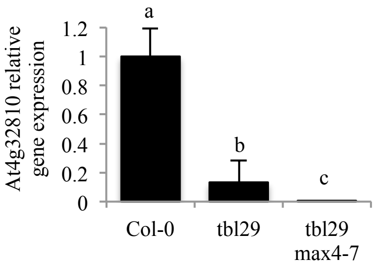

Supplement: Supplementary file 1 [file PLD3-2-e00062-s001.pdf]
