# Supplementary material for: Growth‐ and stress‐related defects associated with wall hypoacetylation are strigolactone‐dependent
Source: Plant Direct. 2018 Jun 13;2(6):e00062. doi: 10.1002/pld3.62 (PMC6508513; doi:10.1002/pld3.62)
Supplement: Supplementary file 2 [file PLD3-2-e00062-s002.pdf]

Supp. Figure 2. Monosaccharide composition, cellulose and acetate content of stem cell walls. Data are represented as mean (AVG) ± the standard deviation (SD) of biological replicates (≥5). Means with different letters are significantly different (Tukey's HSD, $p<0.05$) in the significance (Sig) column.
Fuc=Fucose; Rha=Rhamnose; Ara=Arabinose; Gal=Galactose; Glc=Glucose; Xyl=Xylose; Man=Mannose; GalA; Galacturonic Acid; GlcA= Glucuronic Acid

	**Fuc**			**Rha**			**Ara**			**Gal**			**Glc**			**Xyl**			**Man**			**GalA**			**GlcA**			**Cellulose**			**Acetate**		
	**AVG**	**SD**	**Sig**	**AVG**	**SD**	**Sig**	**AVG**	**SD**	**Sig**	**AVG**	**SD**	**Sig**	**AVG**	**SD**	**Sig**	**AVG**	**SD**	**Sig**	**AVG**	**SD**	**Sig**	**AVG**	**SD**	**Sig**	**AVG**	**SD**	**Sig**	**AVG**	**SD**	**Sig**	**AVG**	**SD**	**Sig**
**Col-0**	1.68	± 0.29	a	8.93	± 1.56	a	5.79	± 0.60	a	12.12	± 0.97	a	25.78	± 3.68	a	99.69	± 4.58	a	10.59	± 1.34	ab	28.98	± 4.17	a	3.77	± 0.46	ab	353.09	± 7.50	a	39.52	± 3.38	a
tbl29	1.45	± 0.03	a	10.20	± 0.38	a	9.07	± 1.28	b	12.65	± 0.56	ab	26.84	± 2.25	a	128.62	± 4.90	b	13.44	± 0.30	b	30.15	± 1.17	a	5.24	± 0.46	b	310.08	± 22.94	b	23.14	± 5.57	b
max4-7	1.71	± 0.18	a	9.11	± 0.81	a	5.90	± 0.58	a	12.05	± 0.72	a	30.90	± 1.52	a	100.46	± 0.35	a	9.69	± 0.24	ab	28.42	± 2.61	a	3.76	± 0.57	a	361.33	± 18.19	a	34.64	± 7.82	a
tbl29S (tbl29 max4-7)	1.80	± 0.10	a	9.37	± 0.37	a	6.48	± 0.29	a	11.49	± 0.37	a	29.99	± 2.93	a	93.95	± 7.32	a	9.22	± 0.64	ab	29.70	± 1.05	a	4.89	± 0.77	b	368.73	± 17.65	a	20.98	± 1.32	b
max4-1	1.87	± 0.16	a	10.02	± 1.11	a	6.66	± 0.93	a	13.21	± 1.30	ab	29.81	± 3.08	a	100.79	± 5.77	a	6.81	± 4.59	a	30.18	± 2.52	a	3.09	± 0.30	a	356.43	± 20.09	a	37.46	± 1.83	a
tbl29 max4-1	1.73	± 0.06	a	10.09	± 0.28	a	7.05	± 0.20	a	12.54	± 0.33	a	25.24	± 2.72	a	98.79	± 6.13	a	9.63	± 0.31	ab	31.26	± 0.62	a	3.97	± 0.36	ab	349.05	± 9.29	a	21.65	± 0.16	b
