# Supplementary material for: Growth‐ and stress‐related defects associated with wall hypoacetylation are strigolactone‐dependent
Source: Plant Direct. 2018 Jun 13;2(6):e00062. doi: 10.1002/pld3.62 (PMC6508513; doi:10.1002/pld3.62)
Supplement: Supplementary file 3 [file PLD3-2-e00062-s003.pdf]

Supp. Figure 3. Monosaccharide composition, cellulose and acetate content of stem cell walls in control and GR24-treated plants growing in hydroponic culture . Data are represented as mean (AVG) ± the standard deviation (SD) of biological replicates (≥5). Means with different letters are significantly different (Tukey’s HSD, $p<0.05$) in the significance (Sig) column. Fuc=Fucose; Rha=Rhamnose; Ara=Arabinose; Gal=Galactose; Glc=Glucose; Xyl=Xylose; Man=Mannose; GalA; Galacturonic Acid; GlcA= Glucuronic Acid

	**Fuc**				**Rha**				**Ara**				**Gal**				**Glc**				**Xyl**			
	**AVG**		**SD**	**Sig**	**AVG**		**SD**	**Sig**	**AVG**		**SD**	**Sig**	**AVG**		**SD**	**Sig**	**AVG**		**SD**	**Sig**	**AVG**		**SD**	**Sig**
Col-0 Control	1.73	±	0.11	ab	9.52	±	0.34	*ac*	8.74	±	0.64	a	16.24	±	0.80	ab	9.48	±	1.04	a	91.39	±	1.97	a
Col-0 GR24	1.51	±	0.12	a	9.17	±	0.48	a	7.60	±	0.21	a	14.50	±	0.29	ac	10.73	±	0.66	a	93.77	±	0.57	a
tbl29 Control	1.83	±	0.02	b	11.45	±	0.27	b	11.71	±	1.60	bc	17.80	±	0.43	b	12.00	±	0.79	ab	97.55	±	1.19	b
tbl29 GR24	1.70	±	0.06	ab	11.69	±	0.28	b	14.61	±	3.00	c	18.31	±	0.67	b	14.60	±	2.07	b	105.21	±	3.68	c
tbl29 max4-7 Control	1.98	±	0.08	b	10.57	±	0.42	bc	9.20	±	0.76	a	16.28	±	0.99	ab	11.11	±	0.58	ab	86.24	±	3.52	a
tbl29 max4-7 GR24	1.48	±	0.06	a	9.02	±	0.31	a	10.86	±	0.47	ab	13.65	±	0.80	c	11.35	±	1.57	ab	97.50	±	1.01	b

	**Man**				**GalA**				**GlcA**				**Cellulose**				**Acetate**			
	**AVG**		**SD**	**Sig**	**AVG**		**SD**	**Sig**	**AVG**		**SD**	**Sig**	**AVG**		**SD**	**Sig**	**AVG**		**SD**	**Sig**
Col-0 Control	9.49	±	1.00	a	2.91	±	0.18	ab	3.84	±	0.34	a	367.52	±	9.80	a	36.44	±	4.16	a
Col-0 GR24	10.98	±	0.51	ab	2.70	±	0.13	a	3.92	±	0.30	ab	344.15	±	8.10	a	36.66	±	5.19	a
tbl29 Control	11.08	±	0.17	ab	3.26	±	0.01	b	5.13	±	0.67	b	318.26	±	13.60	b	19.19	±	1.00	b
tbl29 GR24	12.55	±	0.36	b	3.16	±	0.12	b	5.24	±	0.89	b	309.49	±	9.98	b	18.89	±	1.20	b
tbl29 max4-7 Control	9.72	±	0.47	a	3.12	±	0.08	b	3.79	±	0.44	ab	356.44	±	6.01	a	18.38	±	1.15	b
tbl29 max4-7 GR24	9.71	±	0.17	a	2.61	±	0.17	a	4.86	±	0.44	b	329.49	±	7.42	b	18.88	±	0.76	b
