# Supplementary material for: Growth‐ and stress‐related defects associated with wall hypoacetylation are strigolactone‐dependent
Source: Plant Direct. 2018 Jun 13;2(6):e00062. doi: 10.1002/pld3.62 (PMC6508513; doi:10.1002/pld3.62)
Supplement: Supplementary file 4 [file PLD3-2-e00062-s004.pdf]

	LP	RP	LB
tbl29-1	AATTTGCAAGCAAAGCATCAC	TGGGTTTTTGATAACGAGACG	GCCTTTTCAGAAATGGATAAATA
***max4-7*（*tbl29S*）**	GTATGAGAAACTTCTTGTCGAGGA	GCGAGAGAGAGAAACTGACATTAT	CCGCAATGAGTTGGCTATTCTC
max4-1	TCTTGTGCCCTCTTTCCAATAC	GTCGAAGAAGAGATGCCGGAAAT	TTGTCTCGATGTAGTGGTTGAC
