# Supplementary material for: Growth‐ and stress‐related defects associated with wall hypoacetylation are strigolactone‐dependent
Source: Plant Direct. 2018 Jun 13;2(6):e00062. doi: 10.1002/pld3.62 (PMC6508513; doi:10.1002/pld3.62)
Supplement: Supplementary file 5 [file PLD3-2-e00062-s005.pdf]

Dear Colleen,

Thank you for your kind response. We have now prepared a new version of the manuscript where the reviewer's comments and suggestions have been addressed.
Both reviewers suggest some interesting experiments such as the generation of double mutants in order to study the effect of blocking the SL biosynthesis in other cell wall mutants, or the quantification of SL in other dwarfed mutants. We agree that such exciting experiments are naturally on our future working plan as they would contribute to the question, if the phenomena described in this report are common to other cell wall defects or *tbl29*-specific. In this new version, we added the reviewer's comments to the discussion including these suggested experiments as future work. However, at this stage we think that they are out of the scope of the current manuscript. As the reviewers pointed out a link between cell wall metabolism/ potential sensing and the hormone SL has never been shown before, and this manuscript provides the first solid evidence for this link.
Nevertheless, we found most of the reviewer's suggestions reasonable and helpful and modified the title, abstract, results, discussion and method sections accordingly. We hope you now find the revised manuscript adequate for publication in your journal, Plant Direct.

Reviewer comments:
Reviewer #1:
Major Comments:
• While this is quite an interesting study, I have some lingering mechanistic questions that I feel could be easily resolved and could enhance the findings of the manuscript. The first of these questions is can the max4 mutant suppress other known Arabidopsis mutants with secondary cell wall defects, such as irx1, 3, or 5 mutants involved in cellulose biosynthesis, or the variety of lignin biosynthetic mutants that exhibit collapsed secondary cell walls and other secondary wall-associated phenotypes. I believe that it is important to examine these mutants because it would inform whether the max4 mutant is acting in a xylan acetylation specific manner or if strigolactones are simply responding to general secondary cell wall defects.

We very much agree with the reviewer. The analysis of the effect of *max4* on other irregular xylem mutants, affected in secondary cell wall polymers such as cellulose of lignin, would determine how widespread this principle is. However, we think this is outside of the current scope of this initial report. In the new version of the manuscript we now discuss the experiments suggested by the reviewer as future work in order to further investigate the specificity of the observed mechanism.

• Second, the authors seem to suggest that the genetic lesion in the max4 promoter is causing a transcript reduction that leads to decreased production of the gene product and therefore decreased strigolactone production. I'm not totally convinced that this argument makes sense because the tbl29 mutant has substantially reduced MAX4 transcript

(Supplemental Figure 1B) but exhibits increased strigolactone content (Figure 3). Could it be possible that the genetic lesion in the MAX4 promoter is actually a transcriptional regulatory element that is being disrupted that prevents MAX4 from specifically responding to a TBL29 specific signal? I am sure that figuring out the entire signaling relationship between TBL29 and MAX4 is probably outside the scope of this manuscript, but it seems that this question could be addressed in a relatively straight forward manner.

As the reviewer points out, future work is needed to completely understand the effect of the *max4-7* insertion in the expression of *MAX4*. Although the *max4-7* insertion could be disrupting a transcriptional regulatory element in the *MAX4* promoter, we found a similar suppression effect by using a second *max4* allele (i.e. *max4-1*), where the T-DNA insertion is the 1st intron of the *MAX4* gene (Sorefan et al., 2003). This result indicates that in both cases, the reduced expression of *MAX4* is the cause of the suppression of the *tbl29*-associated defects. As mentioned in the discussion, we attribute the low *MAX4* gene expression level in *tbl29* plants to the higher MeCLA accumulation. A *MAX4* down-regulation has been also observed in plants subjected to phosphate starving where SL synthesis is activated, and upon application of a synthetic strigolactone (Ito et al., 2016; Mashiguchi et al., 2009). Our results agree with the general notion that accumulation of strigolactones triggers the down-regulation of strigolactone biosynthetic genes due to a negative feedback loop observed in several plant species such as *Arabidopsis*, pea or rice (Mashiguchi et al., 2009; Johnson et al., 2006; Arite et al., 2007).
The possibility suggested by the reviewer is now discussed in the new version of the manuscript.

Minor Comments: • The authors should provide more detail about how the genetic material for the initial screen was generated. The authors refer to the max4-7 as an "insertion", which would suggest that the screen was performed using a T-DNA mutant library, but this is not discussed in the results or the materials and methods. Please provide more information regarding how the genetic material for this screen was generated in the results and materials and methods.

In the new version of the manuscript we describe now in the methods section how the genetic material for the mutant screen was generated.

Reviewer #2 :
The authors propose that an unknown mechanism of cell wall damage sensing leads to changes in SL levels, and changes in SL levels somehow cause vessel collapse (Figure 5). However, there is a more obvious interpretation, that a change in plant architecture, caused by SL reduction in max4 mutants, leads to altered transpiration and vascular stress. This is not properly considered. This is surprising, because the earlier esk1 supressor kak1 (Bensussan et al. 2015) showed that increasing stem vasculature can similarly suppress vessel collapse and dwarfing. It is clear to those who grow irx mutants and esk1, that changes in humidity of plant growth changes the extent of vessel collapse and consequently the severity of stress and dark green phenotypes. The authors should consider and discuss fully whether their data can be more simply interpreted as a consequence of altered plant architecture, and altered vessel stress. For example, is the

vessel collapse of esk1 suppressed under low humidity conditions? Is the max4 suppression effective in increased drought? The interpretation is also surprising and not yet convincing because it implies that the physical alterations to the cell wall, caused by defective acetylation of xylan, are themselves not the cause of vessel collapse. I find it hard to understand why the authors propose that xylem vessel collapse could be induced by SL rather than directly by physical weakness under transpiration negative pressures as currently thought.

In the new version of the manuscript we now consider the hypothesis suggested by the reviewer where a change in plant architecture, caused by SL reduction in *max4* mutants, would lead to altered transpiration and vascular stress. However, as mentioned now in the discussion, we consider it unlikely that the reversion of the reduced rosette size and the enhanced freezing tolerance can be explained only by increased stem branching, as these phenotypes are studied when the plants still have no branches. The experiments suggested by the reviewer to determine if the vessel collapse of tbl29 is suppressed under low humidity conditions and if max4 suppression is effective in increased drought conditions are now discussed as future work.

The abstract suggests blocking the biosynthesis of SL is sufficient to recover all the stress- related defects. However, it is difficult to argue this as they have not tested all hormone and transcript levels. Some re-wording is suggested.

In the new version the abstract was modified according to the reviewer's suggestion. The abstract specifies now that blocking MeCLA levels is sufficient to recover *tbl29* defects.

Page 9:" tbl29 shows a 50% increased accumulation of active SLs (i.e. MeCLA) suggesting that SL biosynthesis is altered (Figure 3)". Although statistically significant, can the authors provide evidence that this alteration is specific to this dwarf/stressed mutant? This evidence might be from the literature, or by measuring SLs in other dwarfed mutants affected and unaffected by secondary cell wall stress. Is there evidence that similar changes are not widely seen in plants with altered stature? It is not a profound change.

Plants produce only trace amounts of chemically unstable SLs, which makes it difficult to determine SL contents in plant tissues. Only recently, analytical procedures have been developed, allowing the measurement of a few SL compounds in several plant species. In *Arabidopsis*, MeCLA is active and it has been proposed to be the precursor of other SL (Abe et al., 2014). Unfortunately, the number of mutants tested in the literature is, to our knowledge, limited to genotypes involved in SL biosynthesis and so far, this is the first report showing that a wall deficient mutant shows altered SL levels. We agree with the reviewer that measurement of SL levels on other wall-deficient mutants would be very informative in order to determine if this is a common feature or *tbl29*-specific, but we think this would be outside of the scope of this manuscript. The quantification of SL in other irregular xylem mutants has been included in the new version of the manuscript as a future experiment in order to investigate if an altered SL content is a common feature of

other cell wall defects or *tbl29*-specific.

Minor points: Title: "defects associated to" might be better "defects associated with"

We agree, the title was modified accordingly.

P4: "In addition, the altered shoot system architecture in tbl29 is also rescued, measured as the rosette branching in mature plants (Figure 1C)." This is misleading as it is not just rescue of the reduced stature, but rather increased branching caused by SL reductions.

The sentence was modified accordingly.
